# Curiosity‐based learning in infants: a neurocomputational approach

**DOI:** 10.1111/desc.12629

**Published:** 2017-10-26

**Authors:** Katherine E. Twomey, Gert Westermann

**Affiliations:** ^1^ Division of Human Communication Development and Hearing School of Health Sciences University of Manchester Manchester UK; ^2^ Department of Psychology University of Lancaster Lancaster UK

## Abstract

Infants are curious learners who drive their own cognitive development by imposing structure on their learning environment as they explore. Understanding the mechanisms by which infants structure their own learning is therefore critical to our understanding of development. Here we propose an explicit mechanism for intrinsically motivated information selection that maximizes learning. We first present a neurocomputational model of infant visual category learning, capturing existing empirical data on the role of environmental complexity on learning. Next we “set the model free”, allowing it to select its own stimuli based on a formalization of curiosity and three alternative selection mechanisms. We demonstrate that maximal learning emerges when the model is able to maximize stimulus novelty relative to its internal states, depending on the interaction across learning between the structure of the environment and the plasticity in the learner itself. We discuss the implications of this new curiosity mechanism for both existing computational models of reinforcement learning and for our understanding of this fundamental mechanism in early development.

**RESEARCH HIGHLIGHTS**

- We present a novel formalization of the mechanism underlying infants' curiosity‐driven learning during visual exploration.
- We implement this mechanism in a neural network that captures empirical data from an infant visual categorization task.
- In the same model we test four potential selection mechanisms and show that learning is maximized when the model selects stimuli based on its learning history, its current plasticity and its learning environment.
- The model offers new insight into how infants may drive their own learning.

## 1 INTRODUCTION

For more than half a century, infants' information selection has been documented in lab‐based experiments. These carefully designed, rigorously controlled paradigms allow researchers to isolate a variable of interest while controlling for extraneous environmental influences, offering a fine‐grained picture of the range of factors that affect early learning. Decades of developmental research have brought about a broad consensus that infants' information selection and subsequent learning in empirical tasks are influenced by their existing representations, the learning environment, and discrepancies between the two (for a review, see Mather, 2013). On the one hand, there is substantial evidence that infants' performance in these studies depends heavily on the characteristics of the learning environment. For example, early work demonstrated that infants under 6 months of age prefer to look at patterned over homogenous grey stimuli (Fantz, Ordy, & Udelf, 1962), and in a seminal series of categorization experiments with 3‐month‐old infants, Quinn and colleagues demonstrated that the category representations infants form are directly related to the visual variability of the familiarization stimuli they see (Quinn, Eimas, & Rosenkrantz, 1993; see also Younger, 1985). More recently, 4‐month‐old infants were shown to learn animal categories when familiarized with paired animal images, but not when presented with the same images individually (Oakes, Kovack‐Lesh, & Horst, 2009; see also Kovack‐Lesh & Oakes, 2007). Thus, the representations infants learn depend on bottom‐up perceptual information. Equally, however, infants' existing knowledge has a profound effect on their behavior in these experiments. For example, while newborns respond equivalently to images of faces irrespective of the race of those faces, by 8 months infants show holistic processing of images of faces from their own race, but not of other‐race faces, which they process featurally (Ferguson, Kulkofsky, Cashon, & Casasola, 2009). Similarly, 4‐month‐old infants with pets at home exhibit more sophisticated visual sampling of pet images than infants with no such experience (Hurley, Kovack‐Lesh, & Oakes, 2010; Hurley & Oakes, 2015; Kovack‐Lesh, McMurray, & Oakes, 2014). Effects of learning history also emerge when infants' experience is controlled experimentally. For example, after a week of training with one named and one unnamed novel object, 10‐month‐old infants exhibited increased visual sampling of the previously named object in a subsequent silent looking‐time task (Twomey & Westermann, 2017; see also Bornstein & Mash, 2010; Gliga, Volein, & Csibra, 2010). Thus, learning depends on the interaction between what infants encounter in‐the‐moment and what they know (Thelen & Smith, 1994).

### 1.1 Active learning in curious infants

A long history of experiments, starting with Piaget's (1952) notion of children as “little scientists”, has shown that children are more than passive observers; rather, they take an active role in constructing their own learning. Recent work demonstrates this active learning in infants also. For example, allowing 16‐month‐old infants to choose between two novel objects in an imitation task boosted their imitation of novel actions subsequently performed on the selected item (Begus, Gliga, & Southgate, 2014). Similarly, in a pointing task, 20‐month‐old infants were more likely to elicit help from their caregivers in finding a hidden object when they were unable to see the hiding event than when they saw the object being hidden (Goupil, Romand‐Monnier, & Kouider, 2016). Indeed, even younger infants systematically control their own learning: for example, 7‐ to 8‐month‐olds increased their visual sampling of a sequence of images when those images are moderately—but not maximally or minimally—predictable (Kidd, Piantadosi, & Aslin, 2012; see also Kidd, Piantadosi, & Aslin, 2014). However, as a newly developing field active learning in infants is currently poorly understood (Kidd & Hayden, 2015).

Critically, outside the lab infants interact with their environment freely and largely autonomously, learning about stimuli in whichever order they choose (Oudeyer & Smith, 2016). This exploration is not driven by an external motivation such as finding food to satiate hunger. Rather, it is *intrinsically motivated* (Baldassarre et al., 2014; Berlyne, 1960; Oudeyer & Kaplan, 2007; Schlesinger, 2013): in the real world infants learn based on their own curiosity. Consequently, in constructing their own learning environment, infants shape the knowledge they acquire. However, in the majority of studies on early cognitive development, infants' experience in a learning situation is fully specified by the experimenter, often through a preselected sequence of stimuli that are presented for fixed amounts of time. Thus, we currently know little about the cognitive processes underlying infants' curiosity as a form of intrinsic motivation, or indeed the extent to which what infants learn from curiosity‐driven exploration differs from what they learn in more constrained environments. Given that active exploration is at the heart of development, understanding how they construct their learning experiences—and consequently, their mental representations—is fundamental to our understanding of development more broadly.

### 1.2 Computational studies of intrinsic motivation

In contrast to the relative scarcity of research into infant curiosity, recent years have seen a surge in interest in the role of intrinsic motivation in autonomous computational systems. Equipping artificial learning systems with intrinsic motivation mechanisms is likely to be key to building autonomously intelligent systems (Baranes & Oudeyer, 2013; Oudeyer, Kaplan, & Hafner, 2007), and consequently a rapidly expanding body of computational and robotic work now focuses on the intrinsic motivation mechanisms that may underlie a range of behaviors; for example, low‐level perceptual encoding (Lonini et al., 2013; Schlesinger & Amso, 2013), novelty detection (Marsland, Nehmzow, & Shapiro, 2005), and motion planning (Frank, Leitner, Stollenga, Förster, & Schmidhuber, 2014).

Computational work in intrinsic motivation has suggested a wide range of possible formal mechanisms for artificial curiosity‐based learning (for a review, see Oudeyer & Kaplan, 2007). For example, curiosity could be underpinned by a drive to maximize learning progress by interacting with the environment in a novel manner relative to previously encountered events (Oudeyer et al., 2007). Alternatively, curiosity could be driven by prediction mechanisms, allowing the system to engage in activities for which predictability is maximal (Lefort & Gepperth, 2015) or minimal (Botvinick, Niv, & Barto, 2009). Still other approaches assume that curiosity involves maximizing a system's competence or ability to perform a task (Murakami, Kroger, Birkholz, & Triesch, 2015). Although this computational work investigates numerous potential curiosity algorithms, it remains largely agnostic as to the psychological plausibility of the implementation of those mechanisms (Oudeyer & Kaplan, 2007). For example, many autonomous learning systems employ a separate “reward” module in which the size and timing of the reward are defined a priori by the modeler. Only recently has research highlighted the value of incorporating developmental constraints in curiosity‐based computational and robotic learning systems (Oudeyer & Smith, 2016; Seepanomwan, Caligiore, Cangelosi, & Baldassarre, 2015). While this research shows great promise in incorporating developmentally inspired curiosity‐driven learning mechanisms into artificial learning systems, a mechanism for curiosity in human infants has yet to be specified. The aim of this paper therefore is to develop a theory of curiosity‐based learning in infants, and to implement these principles in a computational model of infant categorization.

### 1.3 The importance of novelty to curiosity‐based learning

From very early in development, infants show a *novelty preference*; that is, they prefer new items to items they have already encountered (Fantz, 1964; Sokolov, 1963). As infants explore an item, however, it becomes less novel; that is, the child *habituates*. During habituation, if a further new stimulus appears, and that stimulus is more novel to the infant than the currently attended item, the infant abandons the habituated item in favor of the new. Thus, novelty and curiosity are linked: broadly, increases in novelty elicit increases in attention and learning (although see e.g., Kidd et al., 2012, 2014, for evidence that excessive novelty leads to a decrease in attention). Here, we propose that curiosity in human infants consists of intrinsically motivated novelty minimization in which discrepancies between stimuli and existing internal representations of those stimuli are optimally reduced (see also Rescorla & Wagner, 1972; Sokolov, 1963).

On this view, infants will selectively attend to stimuli that best support this discrepancy minimization. However, to date there is no agreement in the empirical literature as to what an optimal learning environment might be. For example, Bulf, Johnson, and Valenza (2011) demonstrated that newborns learned from highly predictable sequences of visual stimuli, but not from less predictable sequences. In contrast, 10‐month‐old infants in a categorization task formed a robust category when familiarized with novel stimuli in an order that maximized, but not minimized, overall perceptual differences between successive stimuli (Mather & Plunkett, 2011). Still other studies have uncovered a “Goldilocks” effect in which learning is optimal when stimuli are of intermediate predictability (Kidd et al., 2012, 2014; see also Kinney & Kagan, 1976; Twomey, Ranson, & Horst, 2014). From this perspective, the degree of novelty and/or complexity in the environment that best supports learning is unclear.

Across these studies, novelty and complexity are operationalized differently; for example, as objective environmental predictability (Kidd et al., 2012, 2014), or objective perceptual differences (Mather & Plunkett, 2011). In contrast, in the current work we emphasize that for infants who are engaged in curiosity‐driven learning, novelty is not a fixed environmental quantity but is highly subjective, depending on both perceptual environmental characteristics and what the learner knows. Importantly, each infant has a different learning history which can affect their exploratory behavior. For example, infant A plays with blocks at home and has substantial experience with stacking cube shapes. Infant B's favorite toy is a rattle, and she is familiar with the noise it makes when shaken. Consequently, the blocks at nursery will be more novel to infant B, and the rattle more novel to infant A. On this view, novelty is separate from any objective measure of stimulus complexity; for example, sequence predictability or differences in visual features (Kidd et al., 2012, 2014; Mather & Plunkett, 2011). Thus, a fully specified theory of curiosity‐driven learning must explicitly characterize this subjective novelty based both on the learner's internal representations (what infants know) and the learning environment (what infants experience). In the following paragraphs we provide a mechanistic account of this learner–environment interaction using a neurocomputational model.

### 1.4 Computational mechanisms for infant curiosity

Computational models have been widely used to investigate various cognitive processes, lending themselves in particular to capturing early developmental phenomena such as category learning (e.g., Althaus & Mareschal, 2013; Colunga & Smith, 2003; Gliozzi, Mayor, Hu, & Plunkett, 2009; Mareschal & French, 2000; Mareschal & Thomas, 2007; Munakata & McClelland, 2003; Rogers & McClelland, 2008; Westermann & Mareschal, 2004, 2012, 2014). Here we take a connectionist or neurocomputational approach in which abstract simulations of biological neural networks are used to implement and explore theories of cognitive processes in an explicit way, offering process‐based accounts of known phenomena and generating predictions about novel behaviors. Neurocomputational models employ a network of simple processing units to simulate the learner situated and acting in its environment. Inputs reflect the task environment of interest, and can have important effects across representational development. Like learning in infants, learning in these models emerges from the interaction between learner and environment. Thus, neurocomputational models are well suited to implementing and testing developmental theories.

In the current work we employed *autoencoder* networks: artificial neural networks in which the input and the output are the same (Cottrell & Fleming, 1990; Hinton & Salakhutdinov, 2006; see Figure 2). These models have successfully captured a range of results from infant category learning tasks (Capelier‐Mourguy, Twomey, & Westermann, 2016; French, Mareschal, Mermillod, & Quinn, 2004; Mareschal & French, 2000; Plunkett, Sinha, Møller, & Strandsby, 1992; Westermann & Mareschal, 2004, 2012, 2014). Autoencoders implement Sokolov's (1963) influential account of novelty orienting in which an infant fixates a novel stimulus to compare it with its mental representation. While attending to the stimulus the infant adjusts this internal representation until the two match. At this point the infant looks away from the stimulus, switching attention elsewhere. Therefore, the more novel a stimulus, the longer fixation time will be. Similarly, autoencoder models receive an external stimulus on their input layer, and aim to reproduce this input on the output layer via a hidden layer. Specifically, an input representation is presented to the model via activation of a layer of input nodes. This activation flows through a set of weighted connections to the hidden layer. Inputs to each hidden layer unit are summed and this value passed through a typically sigmoid activation function. The values on the hidden units are then passed through the weighted connections to the output layer. Again, inputs to each output node are summed and passed through the activation function, generating the model's output representation. Learning is achieved by adapting connection weights to minimize error, that is, the discrepancy between the input and output representations. Because multiple iterations of weight adaptation are required to match the model's input and output, error acts as an index of infants' looking times (Mareschal & French, 2000) or, more broadly, the quality of an internal representation.

Self‐supervised autoencoder models are trained with the well‐known generalized delta rule (Rumelhart, Hinton, & Williams, 1986) with the special case that input and target are the same. The weight update rule of these models is:

(1) Δw=η(i−o)o(1−o)

where Δ*w* is the change of a weight after presentation of a stimulus. The first term, (*i − o*), describes the difference between the input and the model's representation of this input. The second term, *o*(*1 − o*), is the derivative of the sigmoid activation function. This term is minimal for output values near 0 or 1 and maximal for *o* = 0.5. Because (*i − o*) represents the discrepancy between the model's input and its representation, and because learning in the model consists of reducing this discrepancy, the size of *o*(1 − *o*) determines the amount the model can learn from a particular stimulus by constraining the size of the discrepancy to be reduced. In this sense, *o*(*1 − o*) reflects the plasticity of the learner, modulating its adaptation to the external environment. Finally, η represents the model's learning rate. The amount of adaptation is thus a function both of the environment and the internal state of the learner.

Because learning in neurocomputational models is driven by the generalized delta rule, we propose that the delta rule can provide a mechanistic account of curiosity‐based learning. Specifically, weight adaptation—learning—is proportional to (*i* − *o*)*o*(*1 − o*); that is, learning is greatest when (*i* − *o*)*o*(*1 − o*) is maximal. If curiosity is a drive to maximize learning, (*i* − *o*)*o*(*1 − o*) offers a mechanism for stimulus selection to maximize learning: a curious model should attempt to maximize its learning by choosing stimuli for which (*i* − *o*)*o*(*1 − o*) is greatest. Below, in Experiment 2 we test this possibility in a model, and compare it against three alternative methods of stimulus selection.

### 1.5 A test case: infant categorization

The ability to categorize—or respond equivalently to—discriminably different aspects of the world is central to human cognition (Bruner, Goodnow, & Austin, 1972). Consequently, the development of this powerful skill has generated a great deal of interest, and a large body of research now demonstrates that infant categorization is flexible and affected by both existing knowledge and in‐the‐moment features of the environment (for a review, see Gershkoff‐Stowe & Rakison, 2005). Categorization therefore lends itself well to testing the curiosity mechanism specified above. In Experiment 1 we present a model that captures infants' behavior in a recent categorization task in which the learning environment was artificially manipulated (thus examining different learning environments in a controlled laboratory study in which infants do not select information themselves). Then, in Experiment 2 we test the curiosity mechanism by “setting the model free”, allowing it to choose its own stimuli. We compare the learner–environment interaction instantiated in the curiosity mechanism against three alternative mechanisms, and demonstrate that learning history and learning plasticity (i.e., the learner's internal state) as well as in‐the‐moment input (i.e., the learning environment) are all necessary for maximal learning. Taken together, these simulations offer an explicit and parsimonious mechanism for curiosity‐driven learning, providing new insight into existing empirical findings, and generating novel, testable predictions for future work.

## 2 EXPERIMENT 1

Early evidence for infants' ability to form categories based on small variations in perceptual features came from an influential series of familiarization/novelty preference studies by Barbara Younger (Younger, 1985; Younger & Cohen, 1983, 1986). In this paradigm, infants are familiarized with a series of related stimuli—for example, an infant might see eight images of different cats, for 10 seconds each. Then, infants are presented with two new images side‐by‐side, one of which is a novel member of the just‐seen category, and one of which is out‐of‐category. For example, after familiarization with cats, an infant might see a new cat and a new dog. Based on their novelty preference, if infants look for longer at the out‐of‐category stimulus than the within‐category stimulus the experimenter concludes that they have learned a category during familiarization which excludes the out‐of‐category item. In this example, longer looking at the dog than the cat image would indicate that infants had formed a “cat” category which excluded the novel dog exemplar (and indeed, they do; Quinn et al., 1993)

**Figure 1:**
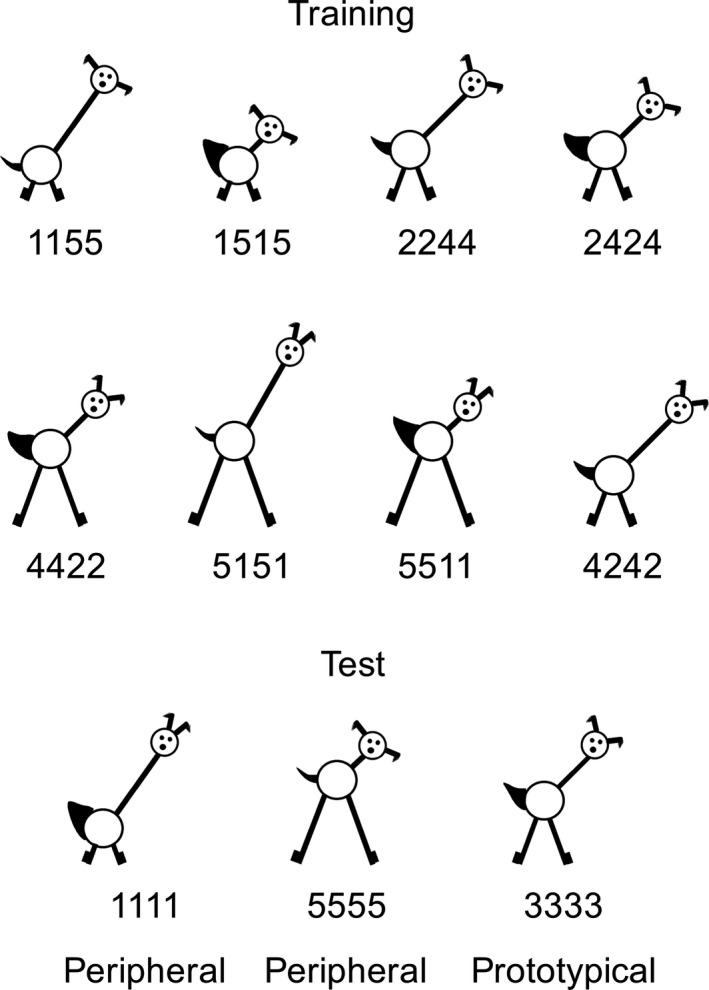
Stimuli used in Younger (1985) and the current simulations. Adapted from Plunkett, Hu & Cohen (2008) and Mather & Plunkett (2011) with permission

Younger (1985) explored whether infants could track covariation of stimulus features and form a category based on this environmental structure. Ten‐month‐old infants were shown a series of pictures of novel animals (see Figure 1) that incorporated four features (ear separation, neck length, leg length and tail width) that could vary systematically in size between discrete values of 1 and 5. At test, all children saw two simultaneously presented stimuli: one peripheral (a new exemplar with extreme feature values) and one category‐central (a new exemplar with the central value for each feature dimension). Infants' increased looking times to the peripheral stimulus indicated that they had learned a category that included the category‐central stimulus. This study was one of the first to demonstrate the now much‐replicated finding that infants' categorization is highly sensitive to perceptual variability (e.g., Horst, Oakes, & Madole, 2005; Kovack‐Lesh & Oakes, 2007; Quinn et al., 1993; Rakison, 2004; Rakison & Butterworth, 1998; Younger & Cohen, 1986).

The target empirical data for the first simulation are from a recent extension of this study which to our knowledge has not yet been captured in a computational model. Mather and Plunkett (2011; henceforth M&P) explored whether the order in which a single set of stimuli was presented during familiarization would affect infants' categorization. They trained 48 10‐month‐old infants with the eight stimuli from Younger (1985, E1). Although all infants saw the same stimuli, M&P manipulated the order in which stimuli were presented during the familiarization phase so that in one condition, infants saw a presentation order which maximized perceptual differences across the stimulus set, and a second condition which minimized overall perceptual differences. At test, all infants saw two simultaneously presented novel stimuli, in line with Younger (1985): one category‐central and one peripheral. M&P found that infants in the maximum distance condition showed an above‐chance preference for the peripheral stimulus, while infants in the minimum distance condition showed no preference. Thus, only infants in the maximum distance condition formed a category.

M&P theorized that if stimuli in this task were represented in a “category space”, then infants in the maximum distance condition would traverse greater distances during familiarization than infants in the minimum distance condition, leading to better learning. However, it is not clear from these empirical data how infants adjusted their representations according to the different presentation regimes. To translate this theory into mechanism, we used an autoencoder network to simulate M&P's task. Closely following the original experimental design, we trained our model with stimulus sets in which presentation order maximized and minimized successive perceptual distances. To enable more fine‐grained analyses we tested additional conditions with intermediate perceptual distances as well as randomly presented sequences (the usual case in familiarization/novelty preference studies with infants). Like M&P we then tested the model on new peripheral and category‐central stimuli. Based on their results, we expected the model to form the strongest category after training with maximum distance stimuli, then intermediate/random distance, and finally minimum distance.

### 2.1 Model architecture

**Figure 2:**
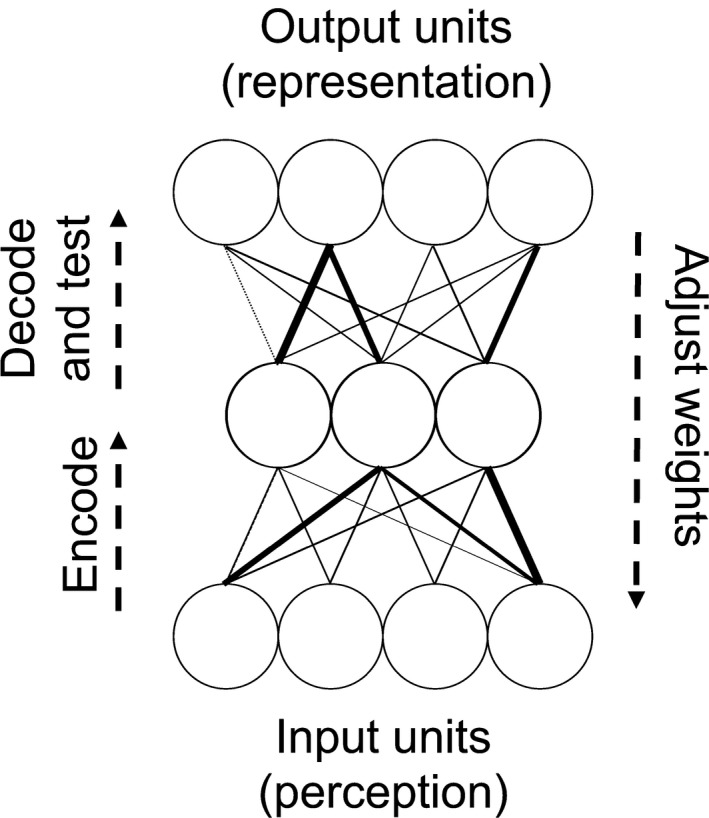
Model architecture

We used an autoencoder architecture consisting of four input units, three hidden units, and four output units (Figure 2). Each input unit corresponded to one of the four features of the training stimuli (i.e., leg length, neck length, tail thickness and ear separation; see Figure 1). Hidden and output units used a sigmoidal activation function and weights were initialized randomly.

### 2.2 Stimuli

Stimuli were based on Younger's (1985) animal drawings with the four features *neck length*,* leg length*,* ear separation*, and *tail width*. Individual stimuli were based on the stimulus dimensions provided in Younger (1985, E1, Broad; see Figure 1). For each feature, these values were normalized to lie between 0 and 1. Each stimulus (that is, input or *i*) therefore consisted of a four‐element vector in which each element represented the value for one of the four features. Model inputs were generated in an identical manner to the stimulus orders used by M&P. We calculated all possible permutations of presentation sequence of the eight stimuli, resulting in 40,320 sequences. In line with M&P, for each sequence we calculated the mean Euclidean distance (ED) between successive stimuli. This resulted in a single overall perceptual distance value for each sequence.

We created orders for the following four conditions based on mean ED:

- Maximum distance (*max*; cf. M&P maximum distance): 24 sets with the largest mean ED
- Minimum distance (*min*; cf. M&P minimum distance): 24 sets with the smallest mean ED
- Medium distance (*med*): 24 sets with an intermediate mean ED, specifically sets 20,149–20,172 when sets are sorted in order of distance (set 20160 is the “median” set)
- stimuli presented in random order

Test sets were identical across conditions, and as in M&P consisted of two category‐peripheral stimuli (new exemplars with extreme feature values) and one category‐central stimulus (a new exemplar with the central value for each feature dimension; see Figure 1). Neither of these test stimuli was part of the training set.

### 2.3 Procedure

During training, each stimulus was presented for a maximum of 20 sweeps (weight updates) or until network error fell below a threshold of 0.01 (Mareschal & French, 2000). The threshold simulated infants' looking away after fully encoding the present stimulus. To obtain an index of familiarization, we tested the model with the entire training set after each sweep (with no weight updating) and recorded sum squared error (*SSE*) as a proxy for looking time (Mareschal & French, 2000; Westermann & Mareschal, 2012, 2014). Order of presentation of training stimuli varied by condition (see Stimuli). Following M&P, we tested the model with three novel test stimuli (two peripheral, one central), presented sequentially for a single sweep with no weight updates, and again recorded SSE. There were 24 separate models in each condition, reflecting the 24 participants in each condition of M&P.

### 2.4 Results and discussion

#### 2.4.1 Training trials

During familiarization infants in M&P demonstrated a significant decrease in looking from the first to the final three‐trial block. For the max and min conditions we submitted SSE during the first and final three‐trial blocks to a 2 (block: first, last; within‐subjects) × 2 (condition: max, min; between‐subjects) mixed ANOVA. In line with M&P, a main effect of block (*F*(1, 46) = 97.35, *p* < .0001, η^2^_G_ = .46) confirmed that overall SSE decreased from the first block (*M* = 0.57, *SD* = 0.11) to the final block (*M* = 0.54, *SD* = 0.11). A main effect of condition (*F*(1, 46) = 2079.12, *p* < .0001, η^2^_*G*_ = .96) revealed that there was less error overall in the max condition (*M* = 0.45, *SD* = 0.03) than in the min condition (*M* = 0.66, *SD* = 0.03). Finally, there was a significant block‐by‐condition interaction (*F*(1, 46) = 4.40, *p* = .041, η^2^_G_ = .03), which arose from a greater decrease in SSE in the max condition (mean decrease = 0.045) than in the min condition (mean decrease = 0.030). Thus, as with the infants in M&P, “looking” in the model decreased over training.

#### 2.4.2 Test trials

**Figure 3:**
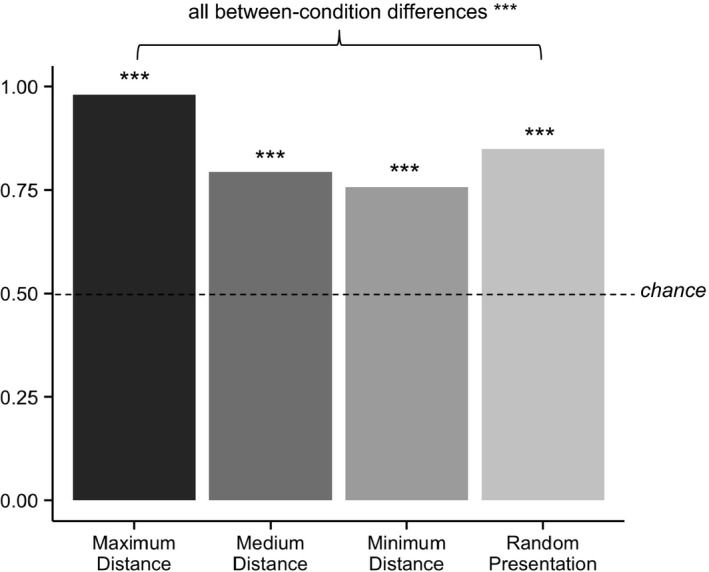
Proportion SSE to peripheral stimulus at test in Experiment 1 ***p < .001

In M&P, increased looking to the peripheral stimuli at test was taken as evidence that infants had learned a category. Again using SSE as a proxy for looking time, we collapsed our analyses across the two peripheral stimuli (Mather & Plunkett, 2011), and calculated proportion of total test SSE (i.e., target looking / target looking + distractor looking) to the peripheral stimulus, as depicted in Figure 3. Wilcoxon rank‐sum tests against chance confirmed that in all conditions the model formed a category (all *V*s = 300, all *p*s < .001). However, a Kruskal‐Wallis test revealed that SSE (and therefore robustness of categorization) differed between conditions (*H*(3) = 80.13, *p* < .001). Post‐hoc Wilcoxon tests (all *W*s two‐tailed and Bonferroni‐corrected) confirmed that the model produced more SSE in the max condition (*Mdn* = 0.99) than in the min condition (*Mdn* = 0.76; *W* = 576, *p* < .0001, *r* = −1.53), the med condition (*Mdn* = 0.79; *W* = 576, *p* < .0001, *r* = −1.53) or the random condition (*Mdn* = 0.83; *W* = 575, *p* < .0001, *r* = −1.51). All other between‐condition differences were also significant (all *p*s < .0001). Note that although infants did not show evidence of category formation in M&P's minimum distance condition, the authors argue that these infants were in fact learning a category; since distances were smaller, these infants traversed less of the category space than their peers in the maximum distance condition, and their category representations were therefore not sufficiently robust to be detected at test. However, our model data are less variable than M&P's empirical data, likely accounting for our detection of differences where M&P found null effects.

Overall, our results support M&P's distance‐based account. We make their theoretical category space explicit by implementing stimuli as feature vectors, which can be interpreted as locations in Euclidean space. The greater overall Euclidean distances in the max condition therefore force the model to “travel” further from trial to trial. Maximizing overall ED leads to greater error early in training, and therefore greater adaptation, resulting in stronger category learning overall. The model therefore explains how manipulation of stimulus order during training can lead to observed differences in learning at test.

In Experiment 1 (as in M&P) the order of stimulus presentation was fixed in each condition to control the mean successive ED. This approach created an artificially structured environment in which the model learned best from the inputs with the most inter‐stimulus variation. Taken together, the empirical and computational data indicate that both infants and the model learn differently in differently structured environments—even when those differences may seem minor, such as the order in which stimuli are experienced. However, Experiment 1 reflected artificially optimized rather than curiosity‐based learning. An important question for research on curiosity‐based learning is how a model that selects its own experiences structures its environment and how learning in this self‐generated environment compares with learning in the artificially optimized environment in Experiment 1. Thus, in Experiment 2 we allowed the model to choose the order in which it learned from stimuli based both on environmental and internal factors. Specifically, in line with theories of intrinsic motivation in which curiosity is triggered when a learner notices a discrepancy between the environment and their representation (e.g., Loewenstein, 1994), the model scans the environment and then selects the stimulus that maximizes a given function. This learning is analogous to an infant looking at and processing an array of objects before choosing one to learn from. We compared the curiosity‐based learning discussed above with three alternative strategies that maximized objective complexity, subjective novelty, or plasticity at each learning step.

## 3 EXPERIMENT 2

In Experiment 2, the model played an active role in its own learning by selecting the order in which it learned from stimuli. We explored four possible mechanisms for stimulus selection.

### 3.1 Model architecture and stimuli

Model architecture and parameters and stimuli were identical to those used in Experiment 1. Stimulus selection proceeded without replacement; thus, as in Experiment 1 the model saw exactly eight stimuli.

### 3.2 Procedure

The procedure used in Experiment 2 was identical to that used in Experiment 1, with the exception that stimulus order was determined by the model based on the following four methods of stimulus selection.

#### 3.2.1 Curiosity

In the *curiosity* condition we tested our formalization of infant curiosity based on the delta rule. Specifically, before presentation of each stimulus, the model calculated (*i − o*)*o*(1 *− o*) for all possible stimuli where *i* = input values and *o* = output values. For example, after presentation of the first stimulus, the model calculated (*i − o*)*o*(1 *− o*) for each of the remaining seven stimuli, resulting in a set of seven potential curiosity values. The next stimulus chosen as input to the model was that for which the absolute value of this curiosity function was maximal. Critically, weights were not updated after this stage, simulating a novelty detection mechanism rather than the novelty reduction process of learning.

#### 3.2.2 Objective complexity maximization

M&P used Euclidean distance as a measure of inter‐stimulus novelty and showed that maximizing novelty objectively present in the learning environment led to better learning than minimizing this novelty. However, M&P selected the presentation orders in advance of the experiment so that the *max* condition maximized mean ED between stimuli across the sequence as a whole. However, our model aimed to provide an account of in‐the‐moment information selection. Thus, in the objective complexity maximization condition, at each step the model chose the stimulus that was maximally distant (by ED) from the *current* stimulus. Complexity is therefore specifically implemented as ED here. In this condition the first stimulus was chosen randomly and successive stimuli were selected so that the next stimulus had the maximal ED (i.e., perceptual distance) from the currently processed stimulus.

#### 3.2.3 Subjective novelty maximization

In the *subjective novelty maximization* condition the model selected stimuli by maximizing *i − o*, leading to the selection of a stimulus that was maximally different from its representation in the model. This mechanism maximized novelty *relative to the model's learning history*. Subjective novelty maximization therefore reflects prediction‐error‐based computational reinforcement learning systems (for a review, see Botvinick et al., 2009; see also Ribas‐Fernandes et al., 2011), in which the learner seeks out learning opportunities that maximize the difference between expectation and observation.

#### 3.2.4 Plasticity maximization

Choosing stimuli based on *o*(1 *− o*) minimizes the in‐the‐moment effect of the environment (*i*) on the model's learning by omitting (*i − o*). Put differently, this mechanism maximizes the model's plasticity. Thus, in the *plasticity maximization* condition the model selected stimuli about which it was most ready to learn (disregarding how much it would actually be able to learn from that stimulus).

In all conditions the test phase was exactly as in Experiment 1, comparing network error to central and peripheral stimuli as a measure of strength of category learning.

### 3.3 Results and discussion

**Figure 4:**
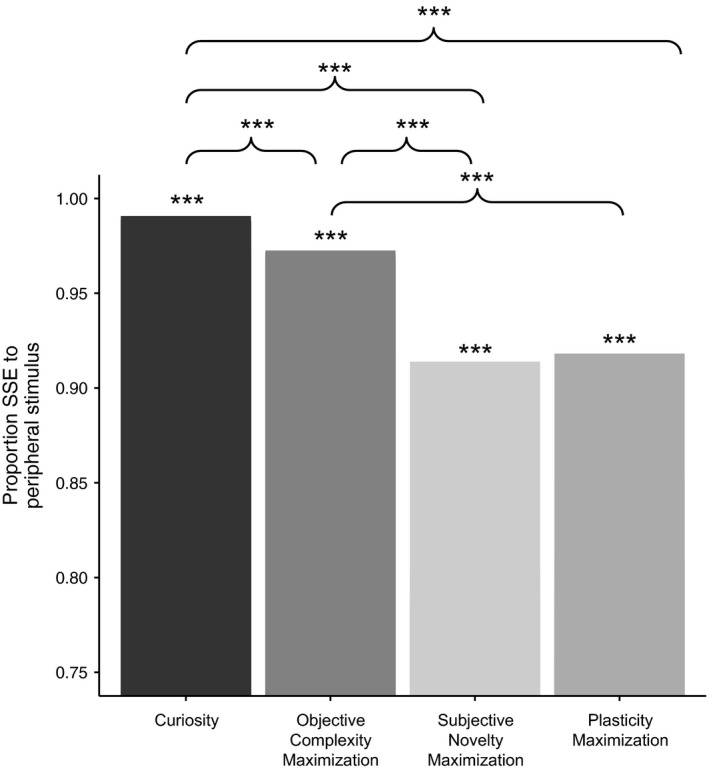
Proportion SSE to peripheral stimulus at test in Experiment 2 ***p < .001

Proportion of total SSE for peripheral test stimuli is depicted in Figure 4. Wilcoxon rank‐sum tests against chance (0.5) confirmed that the model formed a category in all conditions (all *p*s < .001). Active learning therefore led to category formation irrespective of the basis on which the model selected stimuli. A Kruskal‐Wallis test revealed, however, that SSE differed between conditions. In the following section we discuss the differences between the four stimulus selection mechanisms.

Bonferroni‐corrected Wilcox tests confirmed that the model learned best in the curiosity condition. First, the model learned a more robust category in the curiosity condition (*Mdn* = 0.97) than in the objective complexity maximization condition (*Mdn* = 0.91; *W* = 495, *p* < .001, *r* = −0.92). This result highlights the role of the learner in the learning process: when the model selected stimuli based solely on objective, environmental characteristics it learned less well than when it also took into account its own internal state (learning history). The curiosity condition also outperformed the subjective novelty maximization condition (*Mdn* = 0.77; *W* = 575, *p* < .001, *r* = −1.51). Here, although the model's learned representations were taken into account by selecting stimuli for which the difference between its representation (*o*) and the environment (*i*) were greatest in‐the‐moment, the longer‐term effect of learning history, which determines the model's readiness to learn, was ignored. This result demonstrates that the additional plasticity provided by the *o*(1 − *o*) term was necessary for maximal learning; omitting this term affected the extent to which the model could adapt to its learning environment, reducing its ability to select stimuli that would lead to optimum information gain with respect to its learning history. However, maximizing plasticity alone is not sufficient to maximize learning: the model also performed better in the curiosity condition than in the plasticity maximization condition (*Mdn* = 0.75, *W* = 575, *p* < .001, *r* = −1.51). Since this latter mechanism ignores the in‐the‐moment effect of the environment this result suggests that while focusing solely on the environment is not the best strategy for active learning, ignoring how much can actually be learned from a stimulus is not optimal either. Finally, in line with Experiment 1 and M&P, the objective complexity maximization outperformed the subjective novelty and plasticity maximization conditions (respectively, *W* = 564, *p* < .0001, *r* = −1.37; *W* = 56, *p* < .0001, *r* = −1.36), further highlighting the importance of environmental input; however, we found no difference in performance between the subjective novelty maximization and plasticity maximization conditions (*W* = 318, *p* = .55, *r* = −0.12). Overall, then, our formalization of curiosity maximized learning via the dynamic interaction of plasticity, learning history, and in‐the‐moment environmental input.

**Figure 5:**
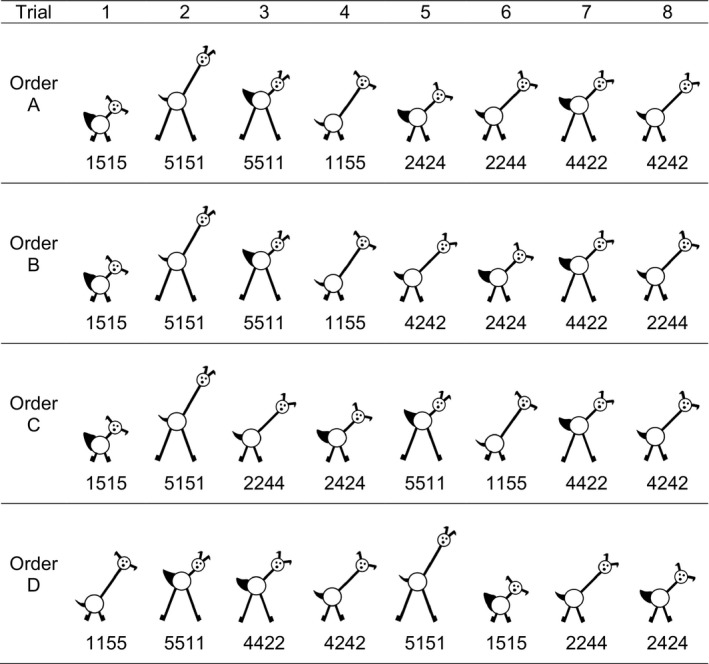
Stimulus orders chosen by curious model

Next, we were interested in the level of complexity of the sequences that maximized learning in the curiosity condition. In the context of Experiment 1 and M&P, we might expect that the curious model had maximized these environmental distances. However, other empirical work suggests that intermediate difficulty could best support learning (Kidd et al., 2012, 2014; Kinney & Kagan, 1976; Twomey et al., 2014). Equally, simplicity has been shown to support learning in some cases (Bulf et al., 2011; Son, Smith, & Goldstone, 2008). To help make sense of these conflicting results, all of which come from experiments with predetermined stimulus presentation orders, we analyzed the stimulus sequences generated by the curious model. Overall, the model generated four different sequences out of the total possible 40,320, depicted in Figure 5. On the one hand, these sequences are very similar; recall that the model selected stimuli without replacement, reducing the degrees of freedom as training proceeded. On the other hand, they are not identical. Their differences stem from the stochasticity provided to the model by the random weight initialization, which can be interpreted as differences between participants (Thomas & Karmiloff‐Smith, 2003). Thus, as in human data, the model data exhibit individual differences underlying a single global pattern of behavior. Nonetheless, since the model generated only four different sequences over 24 runs, this result also predicts that systematicity in infants' curiosity‐based learning should be relatively robust.

**Figure 6:**
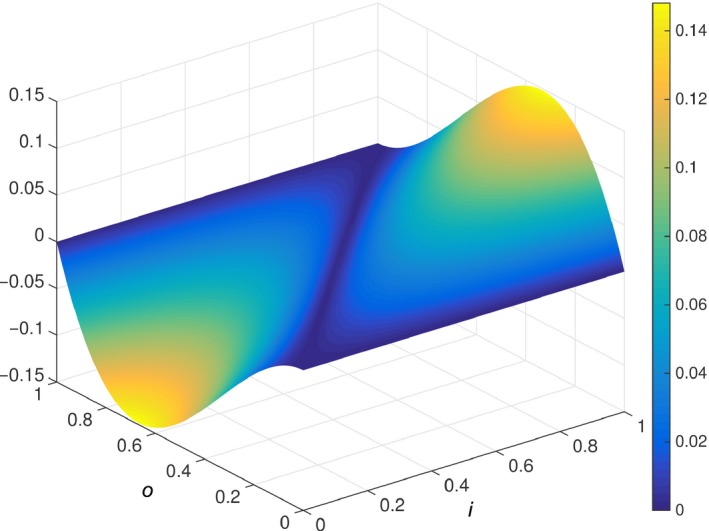
Plot of the curiosity function, (*i − o*)*o*(*1 − o*)

To obtain an index of the level of complexity of the generated orders we ranked the entire set of 40,320 permutations by mean overall ED, generating 281 unique values. Table 1 provides these rankings (higher rank = greater complexity) for the sequences chosen in the curiosity condition. The curious model generated sequences of intermediate objective complexity. However, these sequences were not of average complexity (i.e., from ranks around 140/281) but were towards the high end of the range. To explore this finding we calculated the individual successive EDs for the eight stimuli in each of the four sequences and ranked these according to their complexity (i.e., a rank of 1 would mean that the model has chosen the maximally different next stimulus from the set of remaining stimuli). These individual inter‐stimulus distances are provided in Table 2. Interestingly, the model did not generate intermediate distances at every learning step. Rather, Table 2 illustrates that taking the mean overall ED masks a more interesting behavior: in all sequences, the model first maximized ED (1/7) (cf. M&P). In three out of the four sequences the model then minimized the second ED (6/6), then chose an intermediate ED (3/5) and maximized EDs thereafter. Therefore, when measured in terms of objective complexity, overall intermediate complexity arose from a combination of maximally complex, minimally complex and moderately complex stimuli at different stages of the learning process. Why, then, should optimal intermediacy be shifted towards the more complex end of the scale? Figure 6 plots the curiosity function for values of *i* and *o* between 0 and 1 and illustrates that (*i − o*)*o*(1 *− o*) is minimal when (*i − o*) is zero, and maximal when (*i* − *o*) is around 0.7. Thus, learning is greatest when both plasticity and subjective novelty are intermediate, but shifted towards the higher end of the spectrum.

**Table 1:** Rank mean Euclidean distances chosen in the curiosity condition of Experiment 2

Rank mean ED	Frequency/24
34/281	5
41/281	18
50/281	1

**Table 2:** Euclidean distances (ED) between successive stimuli for sequences chosen in the curiosity condition of Experiment 2

Trial number	Order A (chosen × 1)		Order B (chosen × 5)		Order C (chosen × 11)		Order D (chosen × 7)	
	ED	Rank	ED	Rank	ED	Rank	ED	Rank
1	–	–	–	–	–	–	–	–
2	1.5885	1/7	1.5885	1/7	1.5885	1/7	1.5885	1/7
3	1.0974	3/6	1.0974	3/6	0.3971	6/6	0.3971	6/6
4	1.5885	1/5	1.5885	1/5	0.7942	3/5	0.7942	3/5
5	0.8717	3/4	0.904	2/4	0.904	1/4	0.904	1/4
6	0.5487	3/3	0.7942	1/3	1.5885	1/3	1.5885	1/3
7	0.7942	1/2	0.5742	1/2	1.1914	1/2	1.1914	1/2
8	0.5487	–	0.7942	–	0.7942	–	0.7942	–

This striking novelty‐maximization–novelty‐minimization behavior emerges because curiosity‐driven learning maximizes subjective—not objective—novelty, modulated by the model's plasticity. Specifically, the model is initialized randomly without prior knowledge about the to‐be‐experienced stimuli. At this stage, the stimulus most similar to this random representation in the context of the to‐be‐learned category would be a prototypical, category‐central stimulus. At first, therefore, the model maximizes learning by choosing a category‐peripheral stimulus that is maximally different from its initial, random representation. Next, it chooses the stimulus that again results in maximal subjective novelty—the other category peripheral stimulus. Now, the two most peripheral category stimuli, having just been encoded, are the most familiar to the model and are represented discretely at the extremes of the category space. The stimulus which maximizes subjective novelty should be as equidistant as possible between these two representations; that is, a category‐central stimulus—and this is what the model chooses. Thus, notwithstanding the noise inherent in the initialization of the model, which accounts for its choice of different specific orders, broadly the model explores with a “start from the outside and move in” strategy from the extremes to the prototype. Note that while the model predicts that infants will exhibit the same pattern of exploration this is based on the assumption of no a priori knowledge at the start of learning. Infants, on the other hand, have learned representations by 10 months. Whether infants will exhibit the same pattern of exploration—and whether the pattern holds in different tasks involving truly free exploration—are exciting empirical questions which we are currently addressing.

Why, then, should this pattern maximize learning? In line with the empirical infant categorization literature, if the model generates more error in response to a previously unseen peripheral exemplar relative to a previously unseen prototypical exemplar, we assume that it has learned a category with the prototypical exemplar at its center. In M&P's conceptualization of category learning, exemplars, represented as vectors, can be thought of as locations in representational space. Category learning is therefore a process of moving from location to location within this space. From this perspective, the order in which the curious model chooses stimuli maximizes the number of times it traverses the central location in this space, resulting in strong encoding of this area relative to weak encoding of peripheral stimuli. More generally, the curiosity mechanism makes the intriguing prediction for future work that infants engaged in curiosity‐driven learning will switch systematically between stimuli of maximum and minimum objective complexity.

## 4 GENERAL DISCUSSION

In the current work we used a neurocomputational model to first capture the effect of objective environmental complexity on infants' categorization, and then to offer an explicit account of curiosity‐driven learning in human infants. In Experiment 1 we captured empirical data presented by Mather and Plunkett (2011), in which 10‐month‐old infants formed a robust category when familiarized with stimulus sequences that maximized overall perceptual distance, but not in sequences which minimized it. In Experiment 2, we allowed the model to take an active role in its own learning by letting it select its own stimuli, comparing four different mechanisms for stimulus selection. Here, curiosity‐based learning depended critically on the interaction between learning history, plasticity and the learning environment, allowing the model to choose stimuli for which learning was maximal at the given point of the model's developmental trajectory.

### 4.1 Novelty is in the eye of the beholder

Our goal here was to develop a mechanistic theory of infants' intrinsically motivated—or curiosity‐based—visual exploration. We selected the autoencoder model and its learning mechanism based on their roots in psychological theory and their established success in capturing infants' behavior in empirical tasks. Importantly, the proposed curiosity mechanism is theoretically compatible with classical optimal incongruity approaches (e.g., Hebb, 1949; Kagan, 1972; Loewenstein, 1994; Vygotsky, 1980). According to these theories, learning is optimal in environments of intermediate novelty. Typically, these approaches have interpreted this intermediacy as information that is neither too similar nor too different from what the learner has previously encountered—as seen in the “Goldilocks” effect observed in recent empirical work (Kidd et al., 2012, 2014). Our curiosity mechanism offers a new perspective: what constitutes optimal novelty changes as the child learns. Thus, what is initially too novel to be useful becomes a more suitable input as learning progresses. The model makes this process explicit, choosing stimuli that maximize subjective novelty as modulated by its plasticity. The optimal learning environment is therefore related to subjective novelty, not objective complexity. Critically, this insight may explain the conflicts in the extant literature in which infants in different tasks have been shown to learn best from minimally novel stimuli, maximally novel stimuli, and stimuli of intermediate novelty: the relationship between subjective novelty and objective complexity is nonlinear. That is, different levels of objective complexity could provide an environment of maximal subjective novelty, depending on the infant's learning history. Developing robust methods of tapping subjective novelty in infant looking time tasks, in particular individual differences, is therefore critical to understanding the complex dynamics of early learning.

These simulations offer important predictions for future work in infant curiosity. First, the model shows that based on in‐the‐moment decisions about what aspect of the environment to learn from, learning can be maximal. Given recent work showing that infants can explicitly structure their learning environment by asking their caregivers for help (Goupil et al., 2016), this suggests that infants may also implicitly optimize their own learning (for an early empirical test of this predction, see Twomey, Malem, & Westermann, 2016). Second, in line with looking time studies showing that infants select information systematically (Kidd et al., 2012, 2014), the model chose stimuli of intermediate objective complexity. However, analyses of the sequences chosen by the model predict that rather than seeking out intermediate complexity at each learning event, infants may switch systematically between more and less objectively complex stimuli in the pursuit of maximal subjective novelty. Third, then, our account goes further than classical theories in which curiosity is viewed as either a novelty‐seeking or a novelty‐minimizing behavior (e.g., Loewenstein, 1994). Rather, our model predicts that infants' visual exploration should exhibit both novelty seeking *and* novelty‐minimizing components *when novelty is viewed objectively*, unifying these theories in a single mechanism.

### 4.2 A new approach to computational curiosity in visual exploration

This work contributes to computational research in intrinsic motivation by modeling curiosity using the mechanisms inherent in the existing model based on in‐the‐moment, local decision‐making without a separate, top‐down system for monitoring learning progress and/or reward. Existing computational and robotic systems typically simulate reward as generated by a discrete, engineered module that calculates a reward value using task‐specific computations. Our model departs from this approach, showing that domain‐general mechanisms can produce the motivation to learn, performing a similar function to reward without requiring a separate module; that is, in our model, “reward” is part of the algorithm itself. Overall, then the current work offers an explicit implementation of curiosity in infants' visual exploration, and offers a broader account of the cognitive mechanisms that may drive curiosity: learning that integrates a search for subjective novelty modulated by the learner's plasticity. Here, intrinsically motivated information selection emerges from within the model by exploiting its learning mechanism in a way that optimizes the reduction of discrepancy between expectation and experience.

Overall, this neurocomputational model provides the first formal account of curiosity‐based learning in human infants, integrating subjective novelty and intrinsic motivation mechanisms in a single model. The model is based on the view that early learning is an active process in which infants select information to construct their own optimal learning environment, and it provides a parsimonious mechanism by which this learning can take place. Clearly, our model is restricted to visual exploration; thus, investigating whether these mechanisms generalize to embodied learning situations is an exciting avenue for future work. Equally, it is possible that another one of the many potential mechanisms for intrinsically motivated learning may take over later in development, particularly once metacognition is established and language begins in earnest (e.g., Gottlieb, Oudeyer, Lopes, & Baranes, 2013). Nonetheless, the current implementation of curiosity not only provides novel insight into infant curiosity‐driven category learning and makes predictions for future work both in and outside the lab, but also offers a new mechanistic theory of early intrinsically motivated visual learning.

## References

[desc12629-bib-0001] Althaus, N. , & Mareschal, D. (2013). Modeling cross‐modal interactions in early word learning. IEEE Transactions on Autonomous Mental Development, 5, 288–297.

[desc12629-bib-0002] Baldassarre, G. , Stafford, T. , Mirolli, M. , Redgrave, P. , Ryan, R.M. , & Barto, A. (2014). Intrinsic motivations and open‐ended development in animals, humans, and robots: An overview. Frontiers in Psychology, 5, 985. 25249998 10.3389/fpsyg.2014.00985 PMC4158798

[desc12629-bib-0003] Baranes, A. , & Oudeyer, P.‐Y. (2013). Active learning of inverse models with intrinsically motivated goal exploration in robots. Robotics and Autonomous Systems, 61, 49–73.

[desc12629-bib-0004] Begus, K. , Gliga, T. , & Southgate, V. (2014). Infants learn what they want to learn: Responding to infant pointing leads to superior learning. PLoS ONE, 9, e108817. 25290444 10.1371/journal.pone.0108817 PMC4188542

[desc12629-bib-0005] Berlyne, D.E. (1960). Conflict, arousal, and curiosity. New York: McGraw‐Hill.

[desc12629-bib-0006] Bornstein, M.H. , & Mash, C. (2010). Experience‐based and on‐line categorization of objects in early infancy. Child Development, 81, 884–897. 20573111 10.1111/j.1467-8624.2010.01440.x PMC3167591

[desc12629-bib-0007] Botvinick, M.M. , Niv, Y. , & Barto, A.C. (2009). Hierarchically organized behavior and its neural foundations: A reinforcement learning perspective. Cognition, 113, 262–280. 18926527 10.1016/j.cognition.2008.08.011 PMC2783353

[desc12629-bib-0008] Bruner, J.D. , Goodnow, J.J. , & Austin, G.A. (1972). Categories and cognition In Spradley J.P. (Ed.), Culture and cognition (pp. 168–190). New York: Chandler.

[desc12629-bib-0009] Bulf, H. , Johnson, S.P. , & Valenza, E. (2011). Visual statistical learning in the newborn infant. Cognition, 121, 127–132. 21745660 10.1016/j.cognition.2011.06.010

[desc12629-bib-0010] Capelier‐Mourguy, A. , Twomey, K.E. , & Westermann, G. (2016, August). A neurocomputational model of the effect of learned labels on infants' object representations. Poster presented at the 38th Annual Cognitive Science Society Meeting, Philadelphia, PA.

[desc12629-bib-0011] Colunga, E. , & Smith, L.B. (2003). The emergence of abstract ideas: Evidence from networks and babies. Philosophical Transactions of the Royal Society of London Series B‐Biological Sciences, 358, 1205–1214. 10.1098/rstb.2003.1306 PMC1693214 12903655

[desc12629-bib-0012] Cottrell, G.W. , & Fleming, M. (1990). Face recognition using unsupervised feature extraction In Proceedings of the International Neural Network Conference (pp. 322–325), Paris, France. Dordrecht: Kluwer.

[desc12629-bib-0013] Fantz, R.L. (1964). Visual experience in infants: Decreased attention familiar patterns relative to novel ones. Science, 146, 668–670. 14191712 10.1126/science.146.3644.668

[desc12629-bib-0014] Fantz, R.L. , Ordy, J.M. , & Udelf, M.S. (1962). Maturation of pattern vision in infants during the first six months. Journal of Comparative and Physiological Psychology, 55, 907–917.

[desc12629-bib-0015] Ferguson, K.T. , Kulkofsky, S. , Cashon, C.H. , & Casasola, M. (2009). The development of specialized processing of own‐race faces in infancy. Infancy, 14, 263–284. 10.1080/15250000902839369 32693536

[desc12629-bib-0016] Frank, M. , Leitner, J. , Stollenga, M. , Förster, A. , & Schmidhuber, J. (2014). Curiosity driven reinforcement learning for motion planning on humanoids. Frontiers in Neurorobotics, 7, 25. 24432001 10.3389/fnbot.2013.00025 PMC3881010

[desc12629-bib-0017] French, R.M. , Mareschal, D. , Mermillod, M. , & Quinn, P.C. (2004). The role of bottom‐up processing in perceptual categorization by 3‐ to 4‐month‐old infants: Simulations and data. Journal of Experimental Psychology: General, 133, 382–397. 15355145 10.1037/0096-3445.133.3.382

[desc12629-bib-0018] Gershkoff‐Stowe, L. , & Rakison, D.H. (2005). Building object categories in developmental time. Mahwah, NJ: Psychology Press.

[desc12629-bib-0019] Gliga, T. , Volein, A. , & Csibra, G. (2010). Verbal labels modulate perceptual object processing in 1‐year‐old children. Journal of Cognitive Neuroscience, 22, 2781–2789. 20044900 10.1162/jocn.2010.21427

[desc12629-bib-0020] Gliozzi, V. , Mayor, J. , Hu, J.F. , & Plunkett, K. (2009). Labels as features (not names) for infant categorization: A neurocomputational approach. Cognitive Science, 33, 709–738. 21585482 10.1111/j.1551-6709.2009.01026.x

[desc12629-bib-0021] Gottlieb, J. , Oudeyer, P.‐Y. , Lopes, M. , & Baranes, A. (2013). Information‐seeking, curiosity, and attention: Computational and neural mechanisms. Trends in Cognitive Sciences, 17, 585–593. 24126129 10.1016/j.tics.2013.09.001 PMC4193662

[desc12629-bib-0022] Goupil, L. , Romand‐Monnier, M. , & Kouider, S. (2016). Infants ask for help when they know they don't know. Proceedings of the National Academy of Sciences, USA, 113, 3492–3496. 10.1073/pnas.1515129113 PMC4822620 26951655

[desc12629-bib-0023] Hebb, D. (1949). The organization of behavior: A neuropsychological theory. New York: Wiley.

[desc12629-bib-0024] Hinton, G.E. , & Salakhutdinov, R.R. (2006). Reducing the dimensionality of data with neural networks. Science, 313, 504–507. 16873662 10.1126/science.1127647

[desc12629-bib-0025] Horst, J.S. , Oakes, L.M. , & Madole, K.L. (2005). What does it look like and what can it do? Category structure influences how infants categorize. Child Development, 76, 614–631. 15892782 10.1111/j.1467-8624.2005.00867.x

[desc12629-bib-0026] Hurley, K.B. , Kovack‐Lesh, K.A. , & Oakes, L.M. (2010). The influence of pets on infants' processing of cat and dog images. Infant Behavior and Development, 33, 619–628. 20728223 10.1016/j.infbeh.2010.07.015 PMC2991405

[desc12629-bib-0027] Hurley, K.B. , & Oakes, L.M. (2015). Experience and distribution of attention: Pet exposure and infants' scanning of animal images. Journal of Cognition and Development, 16, 11–30. 25663827 10.1080/15248372.2013.833922 PMC4315258

[desc12629-bib-0028] Kagan, J. (1972). Motives and development. Journal of Personality and Social Psychology, 22, 51–66. 5013358 10.1037/h0032356

[desc12629-bib-0029] Kidd, C. , & Hayden, B.Y. (2015). The psychology and neuroscience of curiosity. Neuron, 88, 449–460. 26539887 10.1016/j.neuron.2015.09.010 PMC4635443

[desc12629-bib-0030] Kidd, C. , Piantadosi, S.T. , & Aslin, R.N. (2012). The Goldilocks effect: Human infants allocate attention to visual sequences that are neither too simple nor too complex. PLoS ONE, 7, e36399. 22649492 10.1371/journal.pone.0036399 PMC3359326

[desc12629-bib-0031] Kidd, C. , Piantadosi, S.T. , & Aslin, R.N. (2014). The Goldilocks effect in infant auditory attention. Child Development, 85, 1795–1804. 24990627 10.1111/cdev.12263 PMC4807134

[desc12629-bib-0032] Kinney, D.K. , & Kagan, J. (1976). Infant attention to auditory discrepancy. Child Development, 47, 155–164. 954493

[desc12629-bib-0033] Kovack‐Lesh, K.A. , McMurray, B. , & Oakes, L.M. (2014). Four‐month‐old infants' visual investigation of cats and dogs: Relations with pet experience and attentional strategy. Developmental Psychology, 50, 402–413. 23731288 10.1037/a0033195

[desc12629-bib-0034] Kovack‐Lesh, K.A. , & Oakes, L.M. (2007). Hold your horses: How exposure to different items influences infant categorization. Journal of Experimental Child Psychology, 98, 69–93. 17604048 10.1016/j.jecp.2007.05.001 PMC2141651

[desc12629-bib-0035] Lefort, M. , & Gepperth, A. (2015). Active learning of local predictable representations with artificial curiosity. Paper presented at the 5th International Conference on Development and Learning and on Epigenetic Robotics, Providence, RI.

[desc12629-bib-0036] Loewenstein, G. (1994). The psychology of curiosity: A review and reinterpretation. Psychological Bulletin, 116, 75–98.

[desc12629-bib-0037] Lonini, L. , Forestier, S. , Teulière, C. , Zhao, Y. , Shi, B.E. , & Triesch, J. (2013). Robust active binocular vision through intrinsically motivated learning. Frontiers in Neurorobotics, 7, 20. 24223552 10.3389/fnbot.2013.00020 PMC3819528

[desc12629-bib-0038] Mareschal, D. , & French, R. (2000). Mechanisms of categorization in infancy. Infancy, 1, 59–76. 10.1207/S15327078IN0101_06 32680314

[desc12629-bib-0039] Mareschal, D. , & Thomas, M.S.C. (2007). Computational modeling in developmental psychology. IEEE Transactions on Evolutionary Computation, 11, 137–150.

[desc12629-bib-0040] Marsland, S. , Nehmzow, U. , & Shapiro, J. (2005). On‐line novelty detection for autonomous mobile robots. Robotics and Autonomous Systems, 51, 191–206.

[desc12629-bib-0041] Mather, E. (2013). Novelty, attention, and challenges for developmental psychology. Frontiers in Psychology, 4, 491. 23914182 10.3389/fpsyg.2013.00491 PMC3730051

[desc12629-bib-0042] Mather, E. , & Plunkett, K. (2011). Same items, different order: Effects of temporal variability on infant categorization. Cognition, 119, 438–447. 21382616 10.1016/j.cognition.2011.02.008

[desc12629-bib-0043] Munakata, Y. , & McClelland, J.L. (2003). Connectionist models of development. Developmental Science, 6, 413–429.

[desc12629-bib-0044] Murakami, M. , Kroger, B. , Birkholz, P. , & Triesch, J. (2015). Seeing [u] aids vocal learning: Babbling and imitation of vowels using a 3D vocal tract model, reinforcement learning, and reservoir computing. Paper presented at the 5th International Conference on Development and Learning and on Epigenetic Robotics, Providence, RI.

[desc12629-bib-0045] Oakes, L.M. , Kovack‐Lesh, K.A. , & Horst, J.S. (2009). Two are better than one: Comparison influences infants' visual recognition memory. Journal of Experimental Child Psychology, 104, 124–131. 18951555 10.1016/j.jecp.2008.09.001 PMC2692647

[desc12629-bib-0046] Oudeyer, P.‐Y. , & Kaplan, F. (2007). What is intrinsic motivation? A typology of computational approaches. Frontiers in Neurorobotics, 1, 6. 18958277 10.3389/neuro.12.006.2007 PMC2533589

[desc12629-bib-0047] Oudeyer, P.‐Y. , Kaplan, F. , & Hafner, V.V. (2007). Intrinsic motivation systems for autonomous mental development. IEEE Transactions on Evolutionary Computation, 11 (2), 265–286.

[desc12629-bib-0048] Oudeyer, P.‐Y. , & Smith, L.B. (2016). How evolution may work through curiosity‐driven developmental process. Topics in Cognitive Science, 8, 492–502. 26969919 10.1111/tops.12196

[desc12629-bib-0049] Piaget, J. (1952). The origins of intelligence in children (Vol. 8). New York: International University Press.

[desc12629-bib-0050] Plunkett, K. , Sinha, C. , Møller, M.F. , & Strandsby, O. (1992). Symbol grounding or the emergence of symbols? Vocabulary growth in children and a connectionist net. Connection Science, 4, 293–312.

[desc12629-bib-0051] Quinn, P.C. , Eimas, P.D. , & Rosenkrantz, S.L. (1993). Evidence for representations of perceptually similar natural categories by 3‐month‐old and 4‐month‐old infants. Perception, 22, 463–475. 8378134 10.1068/p220463

[desc12629-bib-0052] Rakison, D.H. (2004). Infants' sensitivity to correlations between static and dynamic features in a category context. Journal of Experimental Child Psychology, 89, 1–30. 15336916 10.1016/j.jecp.2004.06.001

[desc12629-bib-0053] Rakison, D.H. , & Butterworth, G.E. (1998). Infants' use of object parts in early categorization. Developmental Psychology, 34, 49–62. 9471004 10.1037/0012-1649.34.1.49

[desc12629-bib-0054] Rescorla, R.A. , & Wagner, A.R. (1972). A theory of Pavlovian conditioning: Variations in the effectiveness of reinforcement and nonreinforcement In Black A.H. & Prokasy W.F. (Eds.), Classical Conditioning II: Current Research and Theory (pp. 64–99). New York: Appleton‐Century‐Crofts.

[desc12629-bib-0055] Ribas‐Fernandes, J.J.F. , Solway, A. , Diuk, C. , McGuire, J.T. , Barto, A.G. , Niv, Y. , & Botvinick, M.M. (2011). A neural signature of hierarchical reinforcement learning. Neuron, 71, 370–379. 21791294 10.1016/j.neuron.2011.05.042 PMC3145918

[desc12629-bib-0056] Rogers, T.T. , & McClelland, J.L. (2008). Precis of semantic cognition: A parallel distributed processing approach. Behavioral and Brain Sciences, 31, 689–749.

[desc12629-bib-0057] Rumelhart, D.E. , Hinton, G.E. , & Williams, R.J. (1986). Learning representations by back‐propagating errors. Nature, 323, 533–536.

[desc12629-bib-0058] Schlesinger, M. (2013). Investigating the origins of intrinsic motivation in human infants In Baldassare G. & Mirolli M. (Eds.), Intrinsically motivated learning in natural and artificial systems (pp. 367–392). Berlin: Springer.

[desc12629-bib-0059] Schlesinger, M. , & Amso, D. (2013). Image free‐viewing as intrinsically‐motivated exploration: Estimating the learnability of center‐of‐gaze image samples in infants and adults. Frontiers in Psychology, 4, 802. 24198801 10.3389/fpsyg.2013.00802 PMC3813899

[desc12629-bib-0060] Seepanomwan, K. , Caligiore, D. , Cangelosi, A. , & Baldassarre, G. (2015). The role of intrinsic motivations in the development of tool use: A study in infant robots. Cognitive Processing, 16, S100–S100.

[desc12629-bib-0061] Sokolov, E.N. (1963). Perception and the conditioned reflex. New York: Macmillan.

[desc12629-bib-0062] Son, J.Y. , Smith, L.B. , & Goldstone, R.L. (2008). Simplicity and generalization: Short‐cutting abstraction in children's object categorizations. Cognition, 108, 626–638. 18565504 10.1016/j.cognition.2008.05.002 PMC2584368

[desc12629-bib-0063] Thelen, E. , & Smith, L.B. (1994). A dynamic systems approach to the development of cognition and action. Cambridge, MA: MIT Press.

[desc12629-bib-0064] Thomas, M. , & Karmiloff‐Smith, A. (2003). Connectionist models of development, developmental disorders, and individual differences In Sternberg R.J., Lautrey J. & Lubart T. (Eds.), Models of intelligence: International perspectives (pp. 133–150). Washington, DC: American Psychological Association.

[desc12629-bib-0065] Twomey, K.E. , Malem, B. , & Westermann, G. (2016, May). Infants' information seeking in a category learning task. In In K.E. Twomey (chair), *Understanding infants' curiosity‐based learning: Empirical and computational approaches*. Symposium presented at the XX Biennial International Conference on Infant Studies, New Orleans, LA.

[desc12629-bib-0066] Twomey, K.E. , Ranson, S.L. , & Horst, J.S. (2014). That's more like it: Multiple exemplars facilitate word learning. Infant and Child Development, 23, 105–122.

[desc12629-bib-0067] Twomey, K.E. , & Westermann, G. (2017). Labels shape pre‐speech infants' object representations. Infancy, https://doi.org/10.1111/infa.12201 10.1111/infa.12201 PMC6220954 30450015

[desc12629-bib-0068] Vygotsky, L.S. (1980). Mind in society: The development of higher psychological processes. Cambridge, MA: Harvard University Press.

[desc12629-bib-0069] Westermann, G. , & Mareschal, D. (2004). From parts to wholes: Mechanisms of development in infant visual object processing. Infancy, 5, 131–151. 10.1207/s15327078in0502_2 33401785

[desc12629-bib-0070] Westermann, G. , & Mareschal, D. (2012). Mechanisms of developmental change in infant categorization. Cognitive Development, 27, 367–382.

[desc12629-bib-0071] Westermann, G. , & Mareschal, D. (2014). From perceptual to language‐mediated categorization. Philosophical Transactions of the Royal Society B: Biological Sciences, 369, 20120391. 10.1098/rstb.2012.0391 PMC3866421 24324235

[desc12629-bib-0072] Younger, B.A. (1985). The segregation of items into categories by ten‐month‐old infants. Child Development, 56, 1574–1583. 4075875

[desc12629-bib-0073] Younger, B.A. , & Cohen, L.B. (1983). Infant perception of correlations among attributes. Child Development, 54, 858–867. 6617307

[desc12629-bib-0074] Younger, B.A. , & Cohen, L.B. (1986). Developmental change in infants' perception of correlations among attributes. Child Development, 57, 803–815. 3720405

